# Microbial community analysis reveals high level phylogenetic alterations in the overall gastrointestinal microbiota of diarrhoea-predominant irritable bowel syndrome sufferers

**DOI:** 10.1186/1471-230X-9-95

**Published:** 2009-12-17

**Authors:** Lotta Krogius-Kurikka, Anna Lyra, Erja Malinen, Johannes Aarnikunnas, Jarno Tuimala, Lars Paulin, Harri Mäkivuokko, Kajsa Kajander, Airi Palva

**Affiliations:** 1 Department of Basic Veterinary Sciences, Faculty of Veterinary Medicine, PO Box 66, FI-00014 University of Helsinki, Helsinki, Finland; 2 CSC - Scientific Computing Ltd, Espoo, Finland; 3 DNA Sequencing Laboratory, Institute of Biotechnology, University of Helsinki, Helsinki, Finland; 4 Danisco Innovation, Kantvik, Finland; 5 Valio Ltd, Research Centre, Helsinki, Finland; 6 Department of Biomedicine, Faculty of Medicine, University of Helsinki, Helsinki, Finland; 7 The Finnish Red Cross, Blood Service, Helsinki, Finland

## Abstract

**Background:**

A growing amount of scientific evidence suggests that microbes are involved in the aetiology of irritable bowel syndrome (IBS), and the gastrointestinal (GI) microbiota of individuals suffering from diarrhoea-predominant IBS (IBS-D) is distinguishable from other IBS-subtypes. In our study, the GI microbiota of IBS-D patients was evaluated and compared with healthy controls (HC) by using a high-resolution sequencing method. The method allowed microbial community analysis on all levels of microbial genomic guanine plus cytosine (G+C) content, including high G+C bacteria.

**Methods:**

The collective faecal microbiota composition of ten IBS-D patients was analysed by examining sequences obtained using percent G+C (%G+C) -based profiling and fractioning combined with 16S rRNA gene clone library sequencing of 3267 clones. The IBS-D library was compared with an analogous healthy-control library of 23 subjects. Real-time PCR analysis was used to identify phylotypes belonging to the class *Gammaproteobacteria *and the order *Coriobacteriales*.

**Results:**

Significant differences were found between clone libraries of IBS-D patients and controls. The microbial communities of IBS-D patients were enriched in *Proteobacteria *and *Firmicutes*, but reduced in the number of *Actinobacteria *and *Bacteroidetes *compared to control. In particular, 16S rDNA sequences belonging to the family *Lachnospiraceae *within the phylum *Firmicutes *were in greater abundance in the IBS-D clone library.

**Conclusions:**

In the microbiota of IBS-D sufferers, notable differences were detected among the prominent bacterial phyla (*Firmicutes*, *Actinobacteria*, *Bacteroidetes*, and *Proteobacteria*) localized within the GI tract.

## Background

Irritable bowel syndrome (IBS), a common functional disorder of the gastrointestinal (GI) tract diagnosed in 10-20% of the adult and adolescent populations, is characterized by abdominal pain or discomfort, distorted bowel habits and altered stool characteristics [1]. Although IBS does not predispose to severe ill health, it diminishes the patients' quality of life and has an economic impact on society via work absenteeism and medical leave [2]. Because of the differing symptoms experienced by IBS-diagnosed individuals, sufferers have been divided into four subtypes, i.e. constipation-predominant IBS (IBS-C), diarrhoea-predominant IBS (IBS-D), mixed-type IBS (IBS-M) and unsubtyped IBS [1]. The aetiology of IBS is currently unknown, although genetic, environmental, psychosocial or physiological factors are likely to contribute to the disorder [3,4]. Intestinal bacteria may also play a role in the onset and maintenance of IBS given that previous studies have indicated disparities in the microbiota between IBS-D sufferers and healthy individuals or, to lesser extent, those individuals diagnosed with the other IBS subtypes [5,6]. Consequently, a detailed analysis of the IBS-D associated GI microbiota present is justified.

Several studies have strengthened the argument for a role of intestinal microbes in the causation of IBS. Acute bacterial gastroenteritis often leads to lingering gastrointestinal complaints in individuals, one-third of whom develop IBS [7]. Moreover, elevated levels of serum antibodies specific for bacterial flagellins, the dominant antigens associated with Crohn's disease (CD) [8], have been detected in post-infectious IBS (PI-IBS) sufferers [9]. Evidence for a low level of mucosal inflammation within the GI tract has also emerged for all subtypes of IBS [10,11]. Reports have indicated an increased level of serine protease activity, possibly originating from bacteria [12], in faecal samples recovered from IBS-D sufferers. Although not correlated with bowel movements or present in individuals with acute infectious diarrhoea [13], serine protease activity in mice is associated with increased mucosal colonic permeability and heightened visceral hypersensitivity [12], symptoms that also occur in humans with IBS-D [14,15].

In our study, the combined 16S rRNA gene composition from the faeces of ten individuals with IBS-D, in agreement with the Rome II criteria, was analysed by %G+C profiling and fractioned DNA sequencing, compared with a similarly produced sequence library of 23 healthy individuals [16] and then examined for selected phylotypes using real-time PCR. We observed significant difference between the clone libraries constructed from the IBS-D sufferers and healthy controls samples in the abundance of the four major phyla of bacteria (*Firmicutes*, *Actinobacteria*, *Bacteroidete*s and *Proteobacteria*) found in the GI tract. To the best of our knowledge, these sequencing data represent the first comprehensive study to sufficiently describe the GI microbiota associated with IBS-D.

## Methods

### Study subjects

Faecal samples used for %G+C fractioning and cloning were collected from ten donors (six females, four males), with an average age of 46.5 years that suffered from IBS according to the established Rome II criteria [17]. Real-time PCR assays were performed for two additional individuals diagnosed with IBS-D (one female and one male aged 30 and 27 years, respectively) and for 22 healthy control (HC) individuals that had been used for the construction of the unfractioned DNA sample previously by Krogius-Kurikka *et al*. [16]. IBS-D subjects were excluded from the study if they were pregnant, lactating or unable to cooperate, had consumed antibiotics during the past two months, had undergone major or complicated abdominal surgery, were suffering from organic GI disease, severe systematic disease or endometriosis, or had been diagnosed with senile dementia. Healthy individuals with lactose intolerance, celiac disease and regular GI tract symptoms were not used as controls. All participating IBS-D individuals had undergone clinical and endoscopic GI examinations or had had a barium enema within a year prior to starting the study. Lactose-intolerant IBS-D individuals consuming low-lactose or lactose-free diets as well as individuals medicated for IBS were allowed to participate. All test subjects have been studied previously for a comparison of the GI microbiota between IBS-related and healthy individuals [18-21], and represented the placebo group of a probiotic intervention study [22]. The IBS-D faecal samples were recovered prior to onset of the intervention period and approximately two weeks to 12 months after the clinical GI examination, and the intestinal microbiota contained therein was considered typical of IBS-D sufferers.

### Ethics

The study protocol was approved by the human ethics committee at the joint authority for the District Hospital of Helsinki and the Uusimaa (HUS) region. All participants provided written informed consent and were allowed to withdraw from the study at any point.

### Sequencing of the %G+C profiled sample

To avoid methodological distortions and to enable comparison of IBS-D subjects' microbiota with that of healthy controls', the faecal samples from IBS-D subjects were exposed to the same processes as described in detail by Krogius-Kurikka *et al*. [16]. In brief, the procedure included bacterial genomic DNA isolation with the method described by Apajalahti *et al*. [23], followed by %G+C profiling and fractioning of an equal amount of pooled DNA samples according to their %G+C -content using 5%G+C intervals [24] (Additional file 1). The 16S rRNA was amplified from each of the %G+C fractions 30-35, 35-40, 45-50, 50-55, 60-65, 65-70 and 70-75 separately, with a low number of PCR cycles using two primer pairs with a broad bacterial range [25,26]. The amplicons were pooled and clone libraries were constructed separately for each fraction with the QIAGEN^® ^PCR Cloning plus Kit (Qiagen, Hilden, Germany). The sequencing of the 3' end of the 16S rRNA gene clones was performed with the primer pD' [27]. For templates that were hard to sequence, 1% (v/v) dimethyl sulfoxide was used in the sequencing reaction. These templates were mainly representatives of the phylum *Actinobacteria*.

### Sequence analysis

Sequences were checked manually and the primer sequences were removed with the Staden Package pregap4 version 1.5 and gap version 4.10 assembly programs [28]. Sequences present in more than one clone library were considered to be non-chimeric. Potential chimeras were also searched by manually browsing the ClustalW 1.83 sequence alignment [29] with Bio Edit version 7.0.5.3 [30]. The sequences from %G+C fractions 25-30, 40-45 and 55-60 (AM276372 to AM277303 [21], comprising one-third of the total sample DNA, were included in any further analyses to encompass the whole sample.

### Determination of operational taxonomic units and diversity measurements

MAFFT 6.603b [31] available at the CSC-IT Center for Science (Espoo, Finland) was used for 16S rRNA gene sequence alignments. The MAFFT FFT-NS-2 alignment option was used to align the 16S rRNA gene clone sequences of the IBS-D and healthy control (HC) libraries separately and together. The sequences were cut from the *Escherichia coli *position 430 (totally conserved GTAAA), resulting in an alignment including the 16S rRNA variable regions V1 and V2. The alignments were visually inspected, but they were not edited manually to avoid subjectivity and to maintain reproducibility of the alignments. Distance matrices were created from the cut alignments with Phylip 3.66 Dnadist [32] using the F84 evolution model. The sequences were assigned into phylotypes (operational taxonomic units, OTUs) with DOTUR [33] by applying the furthest neighbor rule in which all sequences within an OTU fulfil the similarity criterion. The 98% cut-off for sequence similarity was used to delimit an OTU. The OTU representatives determined separately for IBS-D and HC libraries were used in the RDP library compare [34] and UniFrac [35] analyses. Common OTU representatives were used in the SONS analysis [36] and in constructing a phylogenetic tree of the family *Lachnospiraceae*. From each OTU with less than 95% similarity to any EMBL nucleotide sequence database entry a representative clone was sequenced to near full-length as described by Krogius-Kurikka *et al*. [16], but the sequence analyses were preformed with the 16S rRNA region covering approximately 450 bp from the 5'-end of the 16S rRNA gene.

The indices for diversity and richness estimates were calculated using DOTUR [33]. Simpson's 1/D and Shannon's indices take into account the number of species present and the abundance of each species, with the value of the indices increasing with greater diversity [37,38]. The Chao estimator for richness [39] considers singletons and doubletons as rare species and the ACE richness estimator considers OTUs represented with less than ten sequences as rare species [40]. Coverage of the clone libraries was calculated with the formula of Good [41], which takes into account the number of singletons in the clone library. The Fasta EMBL Environmental and EMBL Prokaryote database searches [42] and the Ribosomal Database Project II (RDP II) Classifier Tool [43] were used to affiliate the phylotypes.

### Comparison of IBS-D and HC libraries

The 16S rRNA gene clone libraries of the IBS-D patients and healthy controls [16] were compared using SONS [36], RDP library compare [34] and UniFrac [35]. SONS calculates the observed fraction of sequences in shared OTUs in each library and the observed fraction of shared OTUs in each library. A distance matrix calculated based on the common MAFFT alignment of IBS-D and HC sequences, as described above in the *Determination of operational taxonomic units and diversity measurements -*section, was used in the SONS analysis. The RDP library compare online analysis tool was used to make microbial community comparisons and the UniFrac program was applied to compare the microbial communities in a phylogenetic context. In both analyses, a comparison between IBS-D and HC libraries was performed for separately determined OTUs, as described in the section *Determination of operational taxonomic units and diversity measurements*. The RDP analysis was also performed at the sequence level.

In constructing a phylogenetic tree for the UniFrac analysis, a representative sequence of each OTU was aligned with MAFFT [31] using the E-INS-i alignment algorithm. Thereafter, 16S rRNA reference sequences from the European ribosomal RNA database [44] (Additional file 2) were selected and similarly aligned. The MAFFT-profile alignment option was used for constructing a combined profile alignment from the above mentioned alignments. The alignment was cut from *E. coli *position 430 (totally conserved GTAAA) and reference sequences, except for *Methanobrevibacter smithii*, were then deleted from the alignment with BioEdit version 7.0.5.3 [30]. A F84-corrected distance matrix was created using Phylip 3.66 dnadist [32]. The OTU representatives in the tree were labelled with taxonomic information from the Ribosomal Database Project II Classifier Tool [43] to identify the sequence affiliations. UniFrac Significance using abundance weights and P Test Significance analyses were used to describe whether the communities were significantly different overall. Lineage-Specific Analysis was used to test whether significant differences were present between the libraries within separate lineages at a specified distance from the root.

### Phylogenetic tree of the family *Lachnospiraceae*

A phylogenetic tree was constructed for the common OTU representatives from the IBS-D and HC libraries, with over 50% confidence threshold to the family *Lachnospiraceae *(*Firmicutes*) according to the RDP Classifier [43] (Additional file 3). The sequences were aligned with the reference sequences representing the *Clostridium *rRNA XIV group [45] and selected additional reference sequences (Additional files 2 and 3). An MAFFT profile alignment and an F84 distance matrix were constructed as for the UniFrac analysis in the *Comparison of IBS-D and HC libraries *-section, with the exception that sequences from the European ribosomal RNA database representing *Clostridium *rRNA Cluster XIV and *Clostridium leptum *AF262239 were used as references. Bootstrapping the data with one hundred replicates and construction of the tree were performed with the Seqboot and Consense programs of Phylip 3.66 [32]. A phylogenetic tree was generated with a neighbor-joining algorithm from the distance matrix using Phylip 3.66 neighbor [32]. The tree was visualized with MEGA4 [46].

### Real-time PCR assays

**Figure 1:**
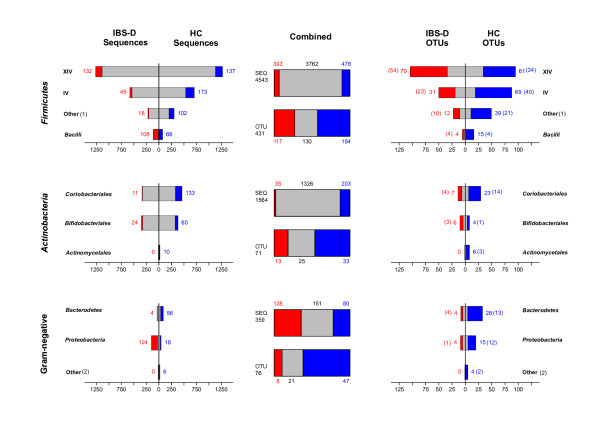
**Distribution of sequences and OTUs among IBS-D and HC clone libraries**. The unique sequences or OTUs (cut-off-level of 98%) in the IBS-D and HC libraries are indicated in red and blue, respectively. The grey area in the group-wise bars is a mirror-image of shared sequences or OTUs and it is presented on both sides of the y-axis. The number of singleton OTUs is given in parentheses. The roman numerals XIV and IV within the *Firmicutes *indicate the corresponding *Clostridium *rRNA clusters, 1) Other *Firmicutes*, 2) *Acidobacteria*, *Cyanobacteria*, TM7 and *Verrucomicrobia*.

The real-time PCR assays for *Enterobacteriaceae *and *Eggerthella lenta *-like phylotypes were developed since the common OTUs for HC and IBS-D, presenting these phylotypes, were notably more abundant in sequences from the IBS-D library (Figure 1) and the genera were significantly more abundant in the IBS-D library according to the RDP library compare tool (Additional file 4). The primers and assays were designed as described in Lyra *et al*. [5]. The forward and reverse primers and positive control clones for real-time PCR assays were 5'-CATAACGTCGCAAGACCAAAGA-3', 5'-GAGTCTGGACCGTGTCTCAGTTC-3' and AM276420 for the first and 5'-GTGACCAACCTGCCCCTTG-3', 5'-GACCCCATCCCTTGCCGT-3' and AM276078 for the latter. The clones AM275716 and AM693283 were used as negative controls for the assays targeting *Enterobacteriaceae *and *Eggerthella lenta *-like phylotypes, respectively. The negative controls were highly similar in sequence with the positive target clones, but included mismatches within the primer annealing sites.

The analyses were performed using the iCycler iQ Real-Time Detection System (Bio-Rad, Hercules, CA, USA) associated with the iCycler Optical System Interface software (version 2.3; Bio-Rad). The 12 IBS-D and 22 HC patients' individual faecal DNA samples were run as triplicates using the following optimized reaction conditions: 25 ng of faecal DNA, 1:75 000 dilution of SYBR Green I (Lonza Biosciences, Basel, Switzerland), 10 mM Tris-HCl (pH 8.8), 50 mM KCl, 0.1% Triton X-100, 2 mM and 3 mM of MgCl_2 _for *Enterobacteriaceae *and *E. lenta *(respectively), 100 μM each dNTP, 0.5 μM each primer, 0.024 U Dynazyme II polymerase (Finnzymes, Espoo, Finland) and 5 μl of either template or water. Standards ranging from 10^2 ^to 10^7 ^16S rRNA gene copies, amplified from the positive controls, were applied. After an initial denaturation at 95°C for 5 min the real-time PCR amplification proceeded with 40 cycles of denaturation at 95°C for 20 s, primer annealing at 64°C for *Enterobacteriaceae *and at 68°C for *E. lenta *for 20 s, extension at 72°C for 30 s and a fluorescence detection step at 85°C for 30 s. A melt curve analysis was preformed after amplification by slow cooling from 95°C to 60°C, with fluorescence collection at 0.3°C intervals and a hold of 10 s at each decrement to check the specificity of the real-time PCR assay. The raw data were transformed to log_10 _ratios of the number of 16S rRNA gene copies in one gram of faeces. The R software environment for statistical computing and graphics [47] was used for performing non-parametric Mann-Whitney U-tests.

### Nucleotide sequence accession numbers

The 16S rRNA gene sequences reported in this study were deposited in the EMBL Nucleotide Sequence Database under accession numbers AM691850 to AM694184.

## Results

### Library characteristics

**Figure 2:**
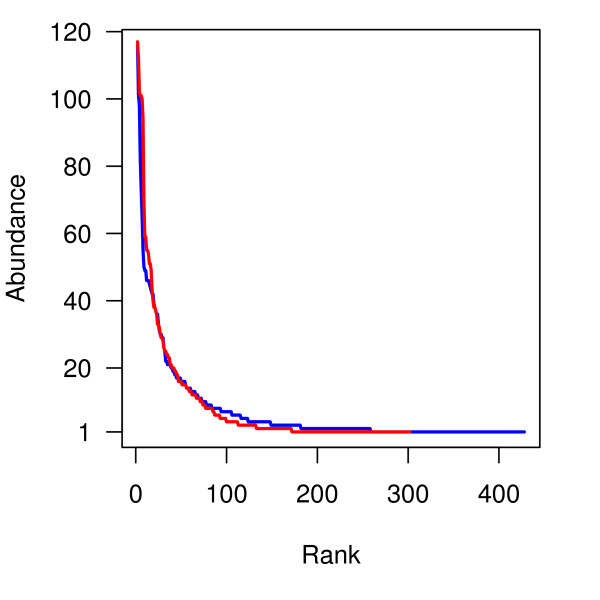
**Rank-abundance plots of the IBS-D and HC libraries**. The curves for IBS-D and HC libraries are indicated in red and blue, respectively. To make the image more compact, the OTUs with the highest number of sequences are deleted (an OTU of 395 sequences for IBS-D and an OTU of 160 sequences for HC).

Approximately 300 sequences were recovered from each %G+C fraction of the IBS-D patients' (n = 10) pooled sample, resulting in a total of 3267 sequences. The IBS-D clone library constructions and sequencing were performed similarly to those of the healthy controls (n = 23) that comprised of 3199 sequences [16] with the exception of amount of subjects pooled to construct the sample (Table 1). According to Good's formula [41], the coverage of clone libraries was above 95%. Less phylotypes were present in the IBS-D library (n = 302) than in the HC library (n = 428), with a 98% cut-off level for OTUs [16] (Table 1). The Shannon and Simpson indices for diversity and Chao and ACE richness estimates were lower for the IBS-D library. The rank abundance curves of the libraries showed highly similar OTU evenness (Figure 2).

**Table 1:** Characteristics of the IBS-D and HC libraries.

**Library characteristic **^**a**^	IBS-D library	**HC library**^**b**^	Combined libraries
No. of subjects in pooled sample	23	10	-

No. of sequences	3267	3199	6466

Average sequence %G+C	55.7	56.2	55.9

No. of OTUs (98%) ^c^	302	428	578

Singletons	131	170	245

Coverage ^d^	96.0	96.2	94.7

Shannon H ^e^	4.463	5.042	5.013

Simpson 1/D ^f^	37.21	81.03	63.10

Chao ^g^	515	613	899

ACE ^h^	533	659	962

a. The library characteristics are based on separate and common alignments created for IBS-D and HC sequences.

b. Sequence data from Krogius-Kurikka *et al*. [16].

c. A 98% cut-off level for sequence similarity was used to delimit OTUs in the analyses.

d. Library coverage according to Good *et al*. [41].

e. Index for diversity [38].

f. Index for diversity [37].

g. Chao-1 richness estimator by Chao [39].

h. Abundance-based coverage estimator by Chao *et al*. [40].

### Microbial community comparisons

**Figure 3:**
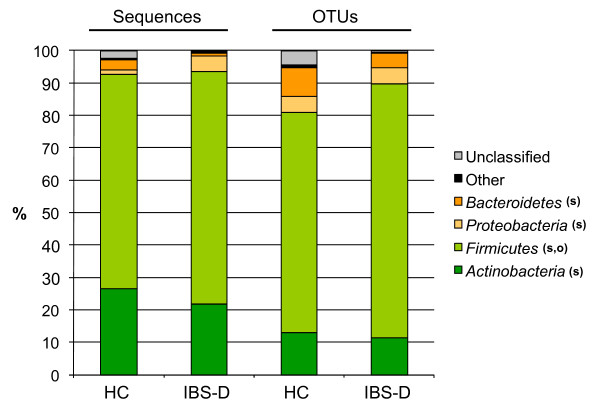
**Relative abundance of phyla in the IBS-D and HC libraries in the RDP library compare analysis **[34]. Significantly differing (p < 0.01) abundances of sequences (s) or OTUs with a cut-off-level of 98% for sequence similarity (o) between the IBS-D and HC libraries are indicated. The "unclassified" phyla have a bootstrap value below 80%.

The microbial community comparison at the phylum level using RDP classifier revealed that the IBS-D library had significantly more representatives of *Proteobacteria *and *Firmicutes *than the HC library, whereas the condition was the opposite with *Actinobacteria *and *Bacteroidetes *(Figure 3). When the comparison was made with OTUs, the IBS-D library was significantly richer in *Firmicutes *than the HC library (Figure 3). In the UniFrac Lineage-Specific Analysis, the phylum *Actinobacteria *differed significantly (p = 0.0013), and the phylum *Bacteroidetes*, the *Firmicutes *families *Lachnospiraceae *and *Ruminococcaeae *and the *Proteobacteria *classes *Gammaproteobacteria *and *Alphaproteobacteria *differed highly significantly (p < 0.001) between the IBS-D and HC libraries. Overall, however, the libraries did not differ significantly.

A substantial proportion of sequences were members of the family *Lachnospiraceae *in both libraries; 45% and 33% in the IBS-D and HC libraries, respectively (Additional file 3). The *Lachnospiraceae *diverged significantly between IBS-D and HC according to the RDP library compare sequence and OTU-based analyses (Additional file 4). Among significantly differing groups of *Actinobacteria*, *Eggerthella *was the only genus more abundant in the IBS-D library than in the HC library (Additional file 4). The IBS-D library contained significantly less *Bacteroidetes *sequences than the HC library (29 vs. 96) in both the RDP library compare and UniFrac analyses. Presence of GI pathogens was not demonstrated by sequencing.

**Figure 4:**
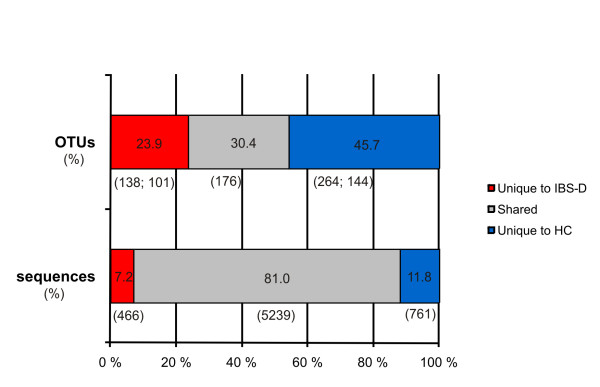
**Percentage of shared and unique OTUs (n = 578) and sequences (n = 6466) in the combined IBS-D and HC libraries**. Number of sequences and OTUs (cut-off-level of 98%) are given in parentheses. The number of singletons is indicated after a semicolon. A common alignment was used for the determination of shared OTUs.

**Figure 5:**
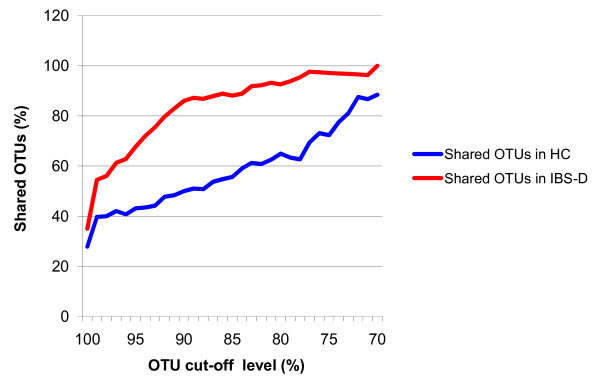
**Abundance of shared OTUs**. Proportion of shared OTUs in the IBS-D and HC libraries according to the SONS analysis [36].

The combined sequence pool from both community samples comprised 578 OTUs, 30.4% of which were shared (Figure 4). The IBS-D library had only half the number of unique OTUs found in the HC library. The majority of the sequences were, however, shared (81.0%), and the proportions of unique sequences in both libraries were similar. The proportion of shared OTUs in the IBS-D library was greater than in the HC library at all OTU cut-off levels, with the highest difference being observed at the (cut-off) level of 90% (Figure 5). The HC library harboured more unique sequences and OTUs than the IBS-D library, with a few exceptions; the portion of unique sequences in the classes *Bacilli *and *Proteobacteria*, and the portion of unique OTUs affiliating with the *Clostridium *XIV cluster and the order *Bifidobacteriales *were more abundant in the IBS-D library (Figure 1).

### Real-time PCR analysis

**Figure 6:**
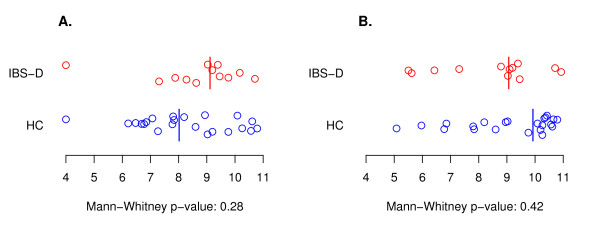
**Real-time PCR results from assays for *Enterobacteriaceae *(A) and *Eggerthella lenta *-like (B) phylotypes**. Samples from IBS-D and HC subjects are in red and blue, respectively. The values are 16S rRNA gene copy numbers per gram of faeces (log_10 _values), and the detection limit is set to 10^4^. Vertical lines are medians from the Mann-Whitney U-test.

The average PCR efficiencies were 89% for the *Enterobacteriaceae *and 96% for *E. lenta *-like real-time PCR assays. The differences between the IBS-D and HC libraries were not significant according to the Mann-Whitney U-tests (Figure 6). The result remained similar when the amounts of detected phylotypes were proportioned to the total amount of bacteria; Mann-Whitney U-test p-values 0.15 and 0.63 for *Enterobacteriaceae *and *Eggerthella lenta *-like phylotypes, respectively (data not shown). According to the dry weights, the moisture content of the samples did not affect the real-time PCR results (data not shown).

## Discussion

To determine whether diarrhoea-predominant IBS is linked to particular changes in the GI microbiota, 16S rRNA gene sequence data constructed from pooled faecal DNA samples of ten IBS patients with diarrhoea were compared with the similarly constructed data of 23 healthy controls [16]. The %G+C fractioning enhances the distinction of a wide spectrum of bacterial phylotypes during the subsequent 16S rRNA gene library construction and sequence analysis. The same extent of cloning was applied to %G+C fractions with varying amount of DNA, both within a sample and between samples, possibly skewing the relative abundance of OTUs detected within the libraries. However, the method allowed thorough comparison of disturbed (IBS-D) and healthy GI microbiota also in the high G+C region.

### Lower number of OTUs in the IBS-D library

The lower diversity and number of OTUs in the IBS-D library is probably mostly due to the smaller number of subjects used to construct the library. On the other hand, a decreased diversity in the GI microbiota of IBS-D patients compared with healthy individuals may exist, as has been observed with CD patients [48]. Acute diarrhoea has been shown to reduce the overall microbiota composition, which could simply be caused by washing out of the commensal bacteria [49]. At the approximate family/genus level (90% OTU cut-off), the number of shared OTUs was above 85% in the IBS-D library compared with 50% in the HC library. Thus the lower diversity of IBS-D patients' GI microbiota seems more apparent at a higher taxonomic level, possibly indicating dysbiosis. A likely cause for these observations is the subject number difference in the pooled samples. On the other hand, the Simpson diversity indexes were very similar below approximately 90% OTU cut-off levels, suggesting little difference between the two libraries (data not shown). However, comparative analysis at higher taxonomic level is likely to be less affected by individuality and thus less prone to such bias.

### Abundance of *Lachnospiraceae *in IBS-D library

Some data suggest an overlap between the aetiology of inflammatory bowel disease (IBD) and IBS [50]. Duck and colleagues [51] isolated a bacterium (A4) with a highly similar flagellin (A4-Fla2) to Fla-X, which has been shown to be an important antigen in CD [8]. The A4 bacterium was classified as a member of the family *Lachnospiraceae *and the *Clostridium *cluster XIVa. Interestingly, elevated antibody concentrations towards the flagellins A4-Fla2 and Fla-X have also been associated with IBS, especially with the PI-IBS subgroup of patients [9], which is most often diarrhoea-predominant [52]. In our study, the family *Lachnospiraceae *was significantly more abundant in the IBS-D library than in the HC library. A large part of the sequences and OTUs in IBS-D (45% and 41%) and HC (33% and 30%) libraries are affiliated with the family *Lachnospiraceae*, including the largest OTU in the IBS-D (n = 395) and HC (n = 160) libraries, which is affiliated with *Eubacterium rectale*, a prevalent member of this family in the gut. Contradictory results on the abundance of *Clostridium coccoides *-*Eubacterium rectale *group bacteria among IBS patients have been obtained in previous studies, which may be due to the different analysis methods used and the broadness of the group [18,20].

Rajilić-Stojanović *et al*. [6] observed a significantly lower level of *Bacteroides *spp. in IBS patients than in healthy controls, but at the *Bacteroidetes *phylum level no difference was detected between the groups. In this study, the number of *Bacteroidetes *phylum sequences was lower in the IBS-D library. However, the number of sequences assigned to this phylum was overall low, and thus, extra caution should be employed in interpreting the data. The group *Bacteroides-Prevotella-Porphyromonas *has previously been quantified from the faeces of healthy controls and IBS patients, with no significant difference between the groups [18]. The maximum four hour delay due to transportation from the study subjects to the laboratory in the freezing of the samples in -70°C may have lowered the proportion of *Bacteroides *detected (Salonen *et al*., personal communications). All samples were, however, stored and processed in the same manner to minimize any technical bias into the comparative analysis.

### Real-time PCR quantification compared with community comparison

The sequencing results were in accordance with the earlier real-time PCR quantification of a *Collinsella aerofaciens *-like phylotype [21], *Bifidobacterium *spp. and *Lactobacillus *spp. [18], all being less abundant in the IBS-D library than in the HC library. Several OTUs comprising at least 100 sequences were not commonly found in the IBS-D library. These OTUs were affiliated with *Bifidobacterium longum *(*Bifidobacteriaceae*), *Bifidobacterium pseudocatenulatum *(*Bifidobacteriaceae*), *Eggerthella lenta *(*Coriobacteriaceae*), a phylotype with 93% similarity to *E. lenta *(*Coriobacteriaceae*), *Enterobacter ludwigii *(*Gammaproteobacteria*) and *Streptococcus bovis *(*Bacillus*). However, real-time PCR assays performed on individual samples for targeting these OTUs, e.g. *B. longum *[18], a *B. catenulatum/B. pseudocatenulatum*-like phylotype [21] and a *S. bovis*-like phylotype [21], have not revealed significant differences between IBS subjects and healthy controls. Additionally, phylotypes affiliating with *Lachnospiraceae *resembling *Ruminococcus torques *with less than 95% 16S rRNA gene sequence similarity have been detected with an association to either IBS-D or healthy depending on the phylotype [5]. Caution should be taken when comparing the real-time PCR results and the sequencing data since the targets of the real-time PCR assays and the taxonomic grouping of the sequence data may not be congruent.

In this study, an *Eggerthella lenta *-like phylotype and an *Enterobacteriaceae *phylotype were quantified with real-time PCR. The median of the 16S rRNA gene quantities for the *E. lenta *-like phylotype was lower for IBS- than for HC (Figure 6), similarly as previously reported for the *Atopobíum *group, which contains the *E. lenta *-like pylotype [18]. However, these differences were not significant. A minor tendency for *Enterobacteriaceae *to be more abundant among IBS-D patients than controls was detected. Consistent with this, elevated number of *Enterobacteria *have previously been detected among CD patients [53]. Evidence of higher quantities of aerobic bacteria in the clone library of IBS-D subjects was seen among *Gammaproteobacteria *and *Bacilli*. An elevated aerobe:anaerobe ratio [19] and higher amounts of *Enterobacteriaceae *[54] have earlier been detected in association with IBS using culture-based techniques. Furthermore, in association with acute diarrhoea, increases in the number of enteric (aerobic) bacteria have been reported [49,55], while anaerobic bacterial counts have decreased [56].

## Conclusions

Based on the 16S rRNA gene sequencing approach applied here, the faecal sample of IBS-D patients showed indications of a dysbiotic microbiota, even though the overall structure was similar to that of healthy controls. Notable differences between IBS-D and controls were identified in all dominant bacterial phyla of the GI microbiota; *Firmicutes*, *Actinobacteria*, *Bacteroidetes *and *Proteobacteria*. Differences on such a high taxonomic level are not as prone to biases in sequence library comparisons as lower level comparisons. Within *Firmicutes*, the family *Lachnospiraceae *was significantly increased in the IBS-D group regarding the number of sequences and OTUs. In future research, the role of this family in IBS-D should receive more attention.

## Competing interests

The authors declare that they have no competing interests.

## Authors' contributions

LK-K and JA edited the sequence data. LK-K and AL conducted the community analyses and together with EM prepared the manuscript. JT acted as a bioinformatics consultant, LP supervised the sequencing process and HM performed the %G+C profiling and fractioning. KK recruited the IBS-D subjects and planned and coordinated the collection of samples. AP coordinated and supervised the study. All authors made corrections and approved the final manuscript.

## Pre-publication history

The pre-publication history for this paper can be accessed here:

http://www.biomedcentral.com/1471-230X/9/95/prepub

## Supplementary Material

Additional file 1 **Percent guanine plus cytosine profile of intestinal microbial genomic DNA pooled from IBS-D (n = 10) and healthy (n = 23) subjects**. The amount of DNA is indicated as relative abundance (%) and the area under the curve is used for calculating the proportional amount of DNA in the separate fractions. The red line indicates IBS-D and the blue line HC. Modified from Kassinen *et al*. [21]. Click here for file

Additional file 2 **RDP reference sequences**. The RDP reference sequences [44] used in the profile alignments for UniFrac analysis [35] and in construction of the phylogenetic tree for the family *Lachnospiraceae*. Roman numerals indicate *Clostridium *rRNA clusters. Click here for file

Additional file 3 **Phylogenetic tree of the family *Lachnospiraceae***. A neighbor-joining tree containing 201 common *Lachnospiraceae *OTUs for IBS-D and HC libraries. The number of sequences within an OTU is denoted after the abbreviation IBS-D or HC. Reference sequences for real-time PCR analyses from the studies by Kassinen *et al*. [21] and Lyra *et al*. [5] and the sequence for bacterium A4 (DQ789118) associated with CD [51] are denoted with red and blue font, respectively. Reference sequences presenting the *Clostridium *rRNA XIV group are denoted with green font. Bootstrap values are percentages of 100 resamplings and the scale bar represents 0.06 substitutions per nucleotide position. Click here for file

Additional file 4 **RDP library compare results for sequences and OTUs**. Significantly differing (p-values < 0.01) groups of sequences and OTUs in the IBS-D and HC libraries and their phylogenetic affiliation according to RDP library compare [34]. The more abundant group is indicated in boldface. Click here for file
