# Supplementary material for: Microbial community analysis reveals high level phylogenetic alterations in the overall gastrointestinal microbiota of diarrhoea-predominant irritable bowel syndrome sufferers
Source: BMC Gastroenterol. 2009 Dec 17;9:95. doi: 10.1186/1471-230X-9-95 (PMC2807867; doi:10.1186/1471-230X-9-95)

Additional file 1 - Percent guanine plus cytosine profile of intestinal microbial genomic DNA pooled from IBS-D (n=10) and healthy (n=23) subjects.

The amount of DNA is indicated as relative abundance (%) and the area under the curve is used for calculating the proportional amount of DNA in the separate fractions. The red line indicates IBS-D and the blue line HC. Modified from Kassinen *et al.* [21].

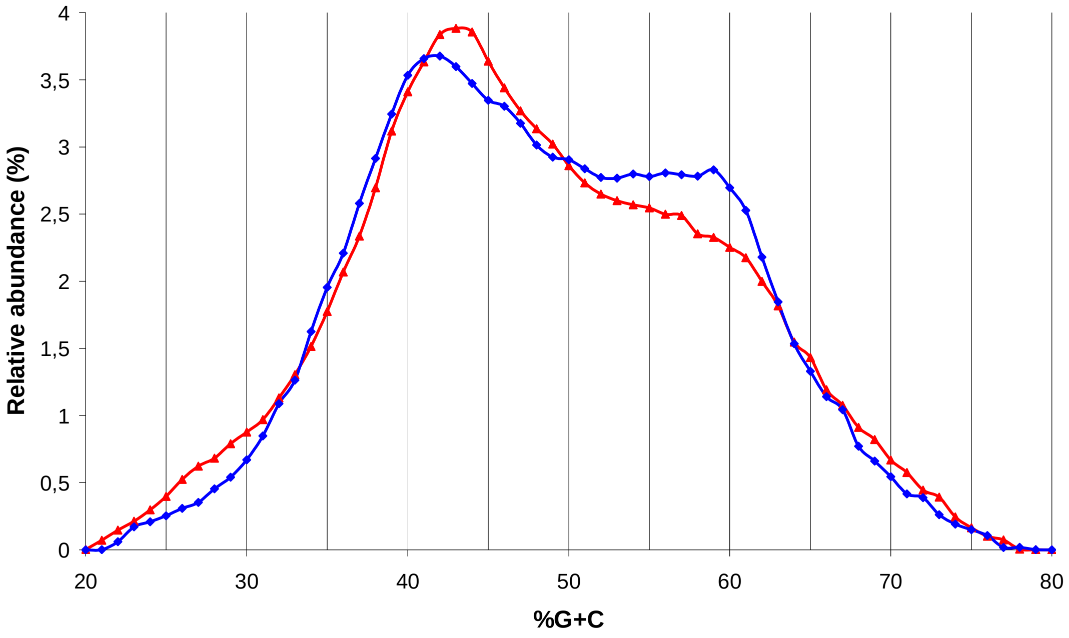

Supplement: Additional file 1 — Percent guanine plus cytosine profile of intestinal microbial genomic DNA pooled from IBS-D (n = 10) and healthy (n = 23) subjects. The amount of DNA is indicated as relative abundance (%) and the area under the curve is used for calculating the proportional amount of DNA in the separate fractions. The red line indicates IBS-D and the blue line HC. Modified from Kassinen et al. [21]. [file 1471-230X-9-95-S1.PDF]
