# Supplementary material for: Microbial community analysis reveals high level phylogenetic alterations in the overall gastrointestinal microbiota of diarrhoea-predominant irritable bowel syndrome sufferers
Source: BMC Gastroenterol. 2009 Dec 17;9:95. doi: 10.1186/1471-230X-9-95 (PMC2807867; doi:10.1186/1471-230X-9-95)
Supplement: Additional file 2 — RDP reference sequences. The RDP reference sequences [44] used in the profile alignments for UniFrac analysis [35] and in construction of the phylogenetic tree for the family Lachnospiraceae. Roman numerals indicate Clostridium rRNA clusters. [file 1471-230X-9-95-S2.PDF]

Additional file 2 - RDB reference sequences.

The RDB reference sequences [44] used in the profile alignments for UniFrac analysis [35] and in construction of the phylogenetic tree for the family *Lachnospiraceae*. Roman numerals indicate *Clostridium* rRNA clusters.

Phylum	Group	Acccession no.	Species of origin
Firmicutes	*Bacilli*	AF104673	*Streptococcus intermedius*
Firmicutes	*Bacilli*	M58802	*Lactobacillus acidophilus*
Firmicutes	*Bacilli*	AB012212	*Enterococcus faecalis*
Firmicutes	*Bacilli*	AF155952	*Bacillus cereus*
Firmicutes	I	M59103	*Clostridium perfringens*
Firmicutes	II	M59094	*Clostridium histolyticum*
Firmicutes	III	X71854	*Clostridium termitidis*
Firmicutes	IV	X85099	*Ruminococcus bromii*
Firmicutes	IV	AF104839	*Ruminococcus albus*
Firmicutes	IV	X85022	*Fusobacterium prausnitzii*
Firmicutes	IV	X81125	*Clostridium viride*
Firmicutes	IV	AF262239	*Clostridium leptum*
Firmicutes	IX	X84005	*Veillonella parvula*
Firmicutes	IX	U95028	*Megasphaera elsdenii*
Firmicutes	V	L09165	*Thermoanaerobacter brockii*
Firmicutes	X	L09183	*Thermoanaerobacter cellulolyticus*
Firmicutes	XI	AF072474	*Clostridium difficile*
Firmicutes	XII	X80833	*Tissierella praeacuta*
Firmicutes	XIII	D14143	*Peptostreptococcus micros*
Firmicutes	XIVa	D14137	Ruminococcus torques
Firmicutes	XIVa	X85101	*Ruminococcus obeum*
Firmicutes	XIVa	L14676	*Roseburia cecicola*
Firmicutes	XIVa	L34627	*Eubacterium rectale*
Firmicutes	XIVa	L76601	*Eubacterium obeum*
Firmicutes	XIVa	L34619	*Eubacterium formicigenerans*
Firmicutes	XIVa	AF067965	*Clostridium methoxybenzovorans*
Firmicutes	XIVa	Y18184	*Clostridium indolis*
Firmicutes	XIVa	D14148	*Coprococcus eutactus*
Firmicutes	XIVa	M59090	*Clostridium coccoides*
Firmicutes	XIX	X77850	*Clostridium rectum*
Firmicutes	XIX	M58683	*Fusobacterium nucleatum*
Firmicutes	XV	M59120	*Eubacterium limosum*
Firmicutes	XVI	M59230	*Eubacterium biforme*
Firmicutes	XVII	M23727	*Lactobacillus vitulinus*
Firmicutes	XVIII	X75908	*Clostridium spiroforme*
Actinobacteria	*Actinomycetales*	AF181690	*Rhodococcus* sp. 5 14
Actinobacteria	*Actinomycetales*	AJ009989	*Propionibacterium freudenreichii*
Actinobacteria	*Actinomycetales*	X79224	*Actinomyces birnadii*
Actinobacteria	*Bifidobacteriales*	D86187	*Bifidobacterium pseudocatenulatum*
Actinobacteria	*Bifidobacteriales*	M58739	*Bifidobacterium longum*
Actinobacteria	*Bifidobacteriales*	D86183	*Bifidobacterium dentium*
Actinobacteria	*Bifidobacteriales*	M58729	*Bifidobacterium adolescentis*
Actinobacteria	*Coriobacteriales*	AF101241	*Slackia heliotrinreducens*
Actinobacteria	*Coriobacteriales*	AF101240	*Slackia exigua*
Actinobacteria	*Coriobacteriales*	AF079507	*Denitrobacterium detoxificans*
Actinobacteria	*Coriobacteriales*	X79048	*Coriobacterium glomerans*
Actinobacteria	*Coriobacteriales*	AB011816	*Eubacterium aerofaciens*
Actinobacteria	*Coriobacteriales*	S44206	*Atopobium parvulum*
Proteobacteria	*Alphaproteobacteria*	X13695	*Brucella abortus*
Proteobacteria	*Alphaproteobacteria*	AF041446	*Bradyrhizobium* sp.

*Proteobacteri*a	*Betaproteobacteria*	U49756	*Oxalobacter formigenes*
Proteobacteria	*Betaproteobacteria*	L37785	*Sutterella wadsworthensis*
Proteobacteria	*Deltaproteobacteria*	U82813	*Bilophila wadsworthia*
Proteobacteria	*Deltaproteobacteria*	M94280	*Polyangium* sp.
Proteobacteria	*Epsilonproteobacteria*	AL139075	*Campylobacter jejuni*
Proteobacteria	*Gammaproteobacteria*	X67024	*Pseudoalteromonas haloplanktis*
Proteobacteria	*Gammaproteobacteria*	M59155	*Hafnia alvei*
Proteobacteria	*Gammaproteobacteria*	Z83204	*Escherichia coli*
Bacteroidetes	*Bacteroidales*	AB003403	*Prevotella ruminicola*
Bacteroidetes	*Bacteroidales*	L16489	*Bacteroides thetaiotaomicron*
Bacteroidetes	*Bacteroidales*	X83946	*Bacteroides fragilis*
Verrucomicrobia	*Verrucomicrobiales*	AF027005	unidentified *Verrucomicrobium*
Acidobacteria	*Acidobacteriales*	D26171	*Acidobacterium capsulatum*
Archaea	*Methanobacteriales*	AF054208	*Methanobrevibacter smithii*
