# Supplementary material for: Microbial community analysis reveals high level phylogenetic alterations in the overall gastrointestinal microbiota of diarrhoea-predominant irritable bowel syndrome sufferers
Source: BMC Gastroenterol. 2009 Dec 17;9:95. doi: 10.1186/1471-230X-9-95 (PMC2807867; doi:10.1186/1471-230X-9-95)
Supplement: Additional file 3 — Phylogenetic tree of the family Lachnospiraceae. A neighbor-joining tree containing 201 common Lachnospiraceae OTUs for IBS-D and HC libraries. The number of sequences within an OTU is denoted after the abbreviation IBS-D or HC. Reference sequences for real-time PCR analyses from the studies by Kassinen et al. [21] and Lyra et al. [5] and the sequence for bacterium A4 (DQ789118) associated with CD [51] are denoted with red and blue font, respectively. Reference sequences presenting the Clostridium rRNA XIV group are denoted with green font. Bootstrap values are percentages of 100 resamplings and the scale bar represents 0.06 substitutions per nucleotide position. [file 1471-230X-9-95-S3.PDF]

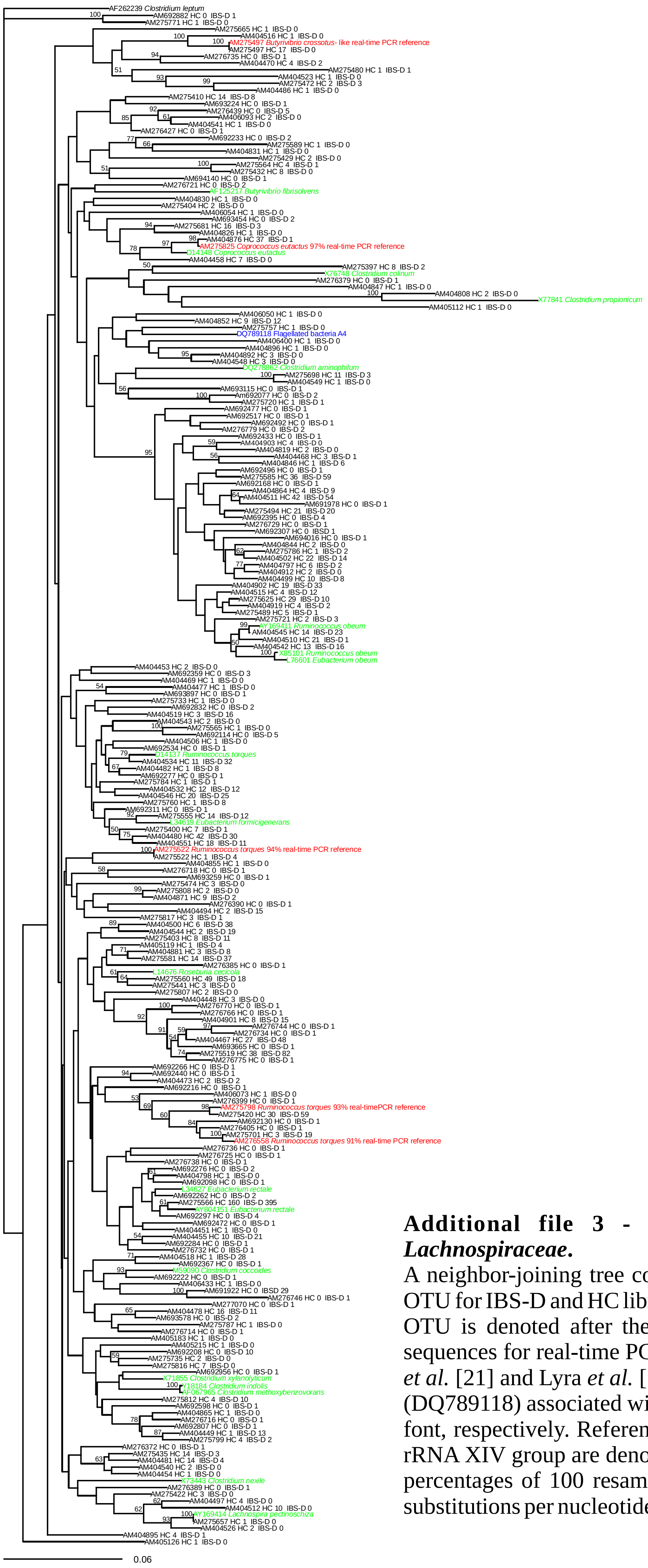

Additional file 3 - Phylogenetic tree of the family *Lachnospiraceae*.

A neighbor-joining tree containing 201 common *Lachnospiraceae* OTU for IBS-D and HC libraries. The number of sequences within an OTU is denoted after the abbreviation IBS-D or HC. Reference sequences for real-time PCR analyses from the studies by Kassinen *et al.* [21] and Lyra *et al.* [5] and the sequence for the bacterium A4 (DQ789118) associated with CD [51] are denoted with red and blue font, respectively. Reference sequences presenting the *Clostridium* rRNA XIV group are denoted with green font. Bootstrap values are percentages of 100 resamplings and the scale bar represents 0.06 substitutions per nucleotide position.
