# Supplementary material for: Microbial community analysis reveals high level phylogenetic alterations in the overall gastrointestinal microbiota of diarrhoea-predominant irritable bowel syndrome sufferers
Source: BMC Gastroenterol. 2009 Dec 17;9:95. doi: 10.1186/1471-230X-9-95 (PMC2807867; doi:10.1186/1471-230X-9-95)
Supplement: Additional file 4 — RDP library compare results for sequences and OTUs. Significantly differing (p-values < 0.01) groups of sequences and OTUs in the IBS-D and HC libraries and their phylogenetic affiliation according to RDP library compare [34]. The more abundant group is indicated in boldface. [file 1471-230X-9-95-S4.PDF]

Additional file 4 - RDP library compare results for sequences and OTUs.

Significantly differing (p <0.01) groups of sequences and OTUs in the IBS-D and HC libraries and their phylogenetic affiliation according to RDP library compare [34]. The more abundant group is indicated in boldface.

RDP classifier taxonomic rank for sequences	IBS-D	HC	p-value
phylum *Proteobacteria*	**155**	42	6.00E-14
class *Gammaproteobacteria*	**130**	8	6.00E-14
order *Enterobacteriales*	**127**	2	4.68E-35
family *Enterobacteriaceae*	**127**	2	4.68E-35
genus *Enterobacter*	**116**	0	4.09E-35
phylum *Actinobacteria*	712	**850**	1.08E-5
class *Actinobacteria*	712	**850**	1.08E-5
genus *Slackia*	3	**18**	7.20E-4
genus *Collinsella*	111	**228**	2.10E-11
genus *Eggerthella*	**233**	144	6.80E-6
subclass *Actinobacteridae*	342	**422**	7.00E-4
order *Actinomycetales*	8	**24**	3.74E-3
order *Bifidobacteriales*	334	**398**	4.96E-3
family *Bifidobacteriaceae*	334	**398**	4.96E-3
genus *Bifidobacterium*	334	**398**	4.96E-3
phylum *Bacteroidetes*	29	**99**	2.99E-10
class *Bacteroidetes*	28	**95**	5.67E-10
order *Bacteroidales*	28	**95**	5.67E-10
family *Bacteroidaceae*	20	**56**	2.67E-5
genus *Bacteroides*	20	**56**	2.67E-5
phylum *Firmicutes*	**2338**	2115	2.60E-6
class *Bacilli*	**111**	61	2.00E-4
order *Lactobacillales*	**106**	59	3.60E-4
family *Streptococcaceae*	**106**	21	6.00E-14
genus *Streptococcus*	**106**	20	6.00E-14
family *Lactobacillaceae*	0	**33**	8.13E-11
genus *Lactobacillus*	0	**31**	3.32E-10
class *Erysipelotrichi*	**124**	78	1.68E-3
order *Erysipelotrichales*	**124**	78	1.68E-3
family *Erysipelotrichaceae*	**124**	78	1.68E-3
genus *Coprobacillus*	**61**	29	9.60E-4
class *Clostridia*	**2098**	1937	2.36E-3
order *Clostridiales*	**2095**	1919	6.00E-4
family Incertae Sedis XIII	0	**7**	7.18E-3
genus *Megasphaera*	**13**	2	4.75E-3
family *Lachnospiraceae*	**1467**	1057	6.00E-14
genus *Coprococcus*	4	**54**	9.88E-13
genus *Lachnosp.* Incertae Sedis	**797**	452	6.00E-14
family *Ruminococcaceae*	525	**709**	5.67E-10
genus *Ruminococcus*	70	**135**	2.60E-6

RDP classifier taxonomic rank for OTUs	IBS-D	HC	p-value
phylum *Firmicutes*	236	**291**	2.52E-3
class *Clostridia*	214	**257**	2.62E-3
order *Clostridiales*	213	**252**	1.28E-3
family *Lachnospiraceae*	**130**	124	8.00E-5
genus *Lachnosp.* Incertae Sedis	**62**	52	2.14E-3
